# Soil Bacterial and Fungal Richness Forecast Patterns of Early Pine Litter Decomposition

**DOI:** 10.3389/fmicb.2020.542220

**Published:** 2020-11-06

**Authors:** Michaeline B. N. Albright, Renee Johansen, Jaron Thompson, Deanna Lopez, La V. Gallegos-Graves, Marie E. Kroeger, Andreas Runde, Rebecca C. Mueller, Alex Washburne, Brian Munsky, Thomas Yoshida, John Dunbar

**Affiliations:** ^1^Biosciences Division, Los Alamos National Laboratory, Los Alamos, NM, United States; ^2^Department of Chemical and Biological Engineering, Colorado State University, Fort Collins, CO, United States; ^3^Center for Biofilm Engineering, Montana State University, Bozeman, MT, United States; ^4^Department of Microbiology and Immunology, Montana State University, Bozeman, MT, United States; ^5^School of Biomedical Engineering, Colorado State University, Fort Collins, CO, United States; ^6^Chemical Diagnostics and Engineering, Los Alamos National Laboratory, Los Alamos, NM, United States

**Keywords:** soil carbon cycling, microbiome, modeling, prediction, community features, litter, pine, machine learning

## Abstract

Discovering widespread microbial processes that drive unexpected variation in carbon cycling may improve modeling and management of soil carbon (Prescott, 2010; Wieder et al., 2015a, 2018). A first step is to identify community features linked to carbon cycle variation. We addressed this challenge using an epidemiological approach with 206 soil communities decomposing Ponderosa pine litter in 618 microcosms. Carbon flow from litter decomposition was measured over a 6-week incubation. Cumulative CO_2_ from microbial respiration varied two-fold among microcosms and dissolved organic carbon (DOC) from litter decomposition varied five-fold, demonstrating large functional variation despite constant environmental conditions where strong selection is expected. To investigate microbial features driving DOC concentration, two microbial community cohorts were delineated as “high” and “low” DOC. For each cohort, communities from the original soils and from the final microcosm communities after the 6-week incubation with litter were taxonomically profiled. A logistic model including total biomass, fungal richness, and bacterial richness measured in the original soils or in the final microcosm communities predicted the DOC cohort with 72 (*P* < 0.05) and 80 (*P* < 0.001) percent accuracy, respectively. The strongest predictors of the DOC cohort were biomass and either fungal richness (in the original soils) or bacterial richness (in the final microcosm communities). Successful forecasting of functional patterns after lengthy community succession in a new environment reveals strong historical contingencies. Forecasting future community function is a key advance beyond correlation of functional variance with end-state community features. The importance of taxon richness—the same feature linked to carbon fate in gut microbiome studies—underscores the need for increased understanding of biotic mechanisms that can shape richness in microbial communities independent of physicochemical conditions.

## Introduction

Modeling existing soil carbon stocks is a starting point to predict future feedbacks to climate (Friedlingstein et al., 2013). Accurate modeling of current carbon stocks remains a challenge as indicated by large unexplained variance, weak spatial correlation at the global scale, and deviation of entire habitat types (Todd-Brown et al., 2013; Wieder et al., 2013; Wang et al., 2017). Many factors may contribute to these discrepancies, but an emerging view posits a strong role for microbial composition (Schimel and Schaeffer, 2012; Wang et al., 2017; Malik et al., 2019; Woolf and Lehmann, 2019) because microbial communities are not always functionally equivalent (Bier et al., 2015). Different microbial community “types” can occur within a habitat type, contributing substantial variation to ecosystem function (Arumugam et al., 2011; Ravel et al., 2011; Falony et al., 2016). A community type is defined as a discernable compositional cluster in a multi-dimensional landscape of compositional possibilities (Arumugam et al., 2011). The existence of alternative soil community types that vary in function under the same environmental conditions has been postulated (Malik et al., 2019), including communities with functional extremes analogous to stable dysbiosis in the human gut (Ochoa-Hueso, 2017). Such communities in nature would create variation in carbon cycling that is unexplained in conventional models.

The specific features of microbial community composition that may drive substantial variation in soil carbon cycling are unknown (Prescott, 2010). Features that have been explored theoretically for effects on litter decomposition rates or soil organic matter abundance include ratios of fungi versus bacteria (Waring et al., 2013), active versus dormant populations (Wang et al., 2017), and oligotrophs versus copiotrophs (Wieder et al., 2015b). However, experimental validation lags (Louis et al., 2016). Microbial diversity has been proposed as a driver of decomposition rates but continues to be intensely debated (Nielsen et al., 2011; Louis et al., 2016) with conflicting experimental evidence against (Degens, 1998; Griffiths et al., 2001; Wertz et al., 2006; de Graaff et al., 2015) and for (Griffiths et al., 2000; Juarez et al., 2013; Maron et al., 2018; Wagg et al., 2019). In recent studies supporting a diversity-decomposition relationship, a single community was manipulated in each case by extreme dilution (e.g., undiluted versus 10^–5^ fold) (Juarez et al., 2013; Maron et al., 2018) or by size-fractionation of a soil (Wagg et al., 2019) to create diversity gradients, but these gradients seem unlikely to occur under natural scenarios. Examining diverse microbial communities in nature that foster different carbon cycling patterns under the same environmental conditions is a useful alternative to discover relevant community features.

Microbial features that shape the quantity and quality of dissolved organic carbon (DOC) from plant litter decomposition are of particular interest (de Graaff et al., 2015). DOC is the mobile pool of soil carbon that can be transported to deeper soil layers where long-term stabilization on mineral surfaces can occur (Kalbitz and Kaiser, 2008). The quantity and quality of DOC influence the amount of carbon that binds to mineral surfaces (Kalbitz and Kaiser, 2008). DOC is released from plant litter by disruption of plant cells containing soluble material and by microbial hydrolysis of complex plant compounds. DOC is also released from microbes via active secretions (e.g., enzymes, antibiotics, signaling molecules), metabolic waste products, and necromass. In theory, variation in microbial community composition can dramatically alter DOC quantity and quality by changing DOC consumption or production rates and the types of compounds preferentially decomposed or added in the DOC pool.

To explore microbial effects on DOC, we used an epidemiological approach wherein a large population of plant litter decomposer communities in laboratory microcosms was screened to delineate cohorts with contrasting DOC concentrations. Although surface leaf litter decomposition is only one component of soil carbon cycling, it accounts for about half of the CO_2_ efflux in temperate deciduous forests annually (Schlesinger and Andrews, 2000). Plant litter decomposition is generally viewed as a two-stage process comprising an initial fast phase dominated by weedy microbial taxa, and a subsequent slow phase driven by taxa better equipped to deconstruct lignocellulose (Cotrufo et al., 2015; Müller et al., 2015). The early phase of litter decomposition is of interest because carbon flow during rapid microbial growth on labile plant carbon is now understood to play an important role in the formation of soil organic matter (Rubino et al., 2010; Cotrufo et al., 2015).

To acquire a spectrum of decomposer communities on Ponderosa pine leaf litter, 206 soil samples were collected from nine states in the southwestern United States (Supplementary Figure S1) as source material for the dispersal of microbial communities onto leaf litter in 618 microcosms. We measured carbon flow during the early phase of plant litter decomposition by quantifying DOC from a 6-week decomposition period. Community cohorts were delineated as “high” versus “low” DOC. We used a simple mineral binding assay *in vitro* to assess potential differences in DOC composition that have implications for soil carbon accumulation. We also measured cumulative carbon dioxide (CO_2_) to assess the degree of variation between CO_2_ and DOC. A strong correlation between CO_2_ and DOC would enable use of CO_2_ as a proxy for DOC, which is a slower and expensive measurement, whereas a weak correlation may suggest variation in controls on each carbon pool, motivating future comparisons of microbial community features driving the abundance of each pool of carbon. We hypothesized that the composition of the original soils would exert legacy effects that constrain succession in each microcosm, and therefore specific community features in the original soils would be linked to the final DOC concentrations in the microcosms.

## Materials and Methods

### Initial Soil Collection for Microbial Inoculum

Soil samples were collected from 206 locations throughout the southwestern United States between February and April, 2015 (Supplementary Figure S1). The goal of this study was not to relate functional outcomes to detailed characteristics of the environments from which the soils were collected. Therefore, a randomized collection scheme was not used, as this would have substantially increased the cost and logistical burden of sample collection without benefit. Samples were typically collected at locations approximately 80 km apart, at least 15 m from roadways, from the top 3 cm of the soil surface after removal of surface litter. In the collection region, ecosystems routinely have patchy ground cover with exposed soil and little, if any, litter layer at the soil surface. Samples were collected in sterile 50-ml screw-cap tubes, and immediately stored on ice. Samples were stored at 6°C in the laboratory to avoid microbial lysis from freeze-thaw effects and were used within 6 weeks to inoculate microcosms. The location of each sample was recorded by GPS and photographed (e.g., Supplementary Figure S1) to facilitate description of the eight major ecosystem types (Table 1) from which samples were obtained. The eight ecosystem types were defined broadly by dominant and minor plant types or by agricultural land-use. GPS coordinates, sample location photos, and ecosystem type for each sample are available upon request.

**TABLE 1:** Prevalence of DOC categories within ecosystem types.

	Samples per DOC category^*c*^		
Ecosystem type^*a*^	Low	Medium	High
Grassland - shrub	23	44	50
Mixed^*b*^	10	8	3
Juniper woodland - grass	10	3	2
Agricultural field, active	3	10	2
Agricultural field, fallow	4	6	3
Grassland - juniper	5	3	2
Pinyon juniper woodland - grass	6	2	0
Pine forest	3	2	2

*^*a*^For the hyphenated ecosystem types, the dominant plant type is listed first. ^*b*^“Mixed” represented locations that were predominantly low-lying drainage areas with highly mixed plant types (trees, shrubs, and grasses). ^*c*^The frequency of samples in “high” or “low” categories differed significantly from chance (*X*^2^ test, d.f. = 2, *X*^2^ = 17.89, *P* < 0.001). For this test, data were used only from the first three ecosystem types, which were adequately sampled such that the expected frequency of high or low DOC samples was greater than 5.*

### Microcosm Construction and CO_2_ Sampling

Microcosms consisted of 125 ml serum bottles containing approximately 5 g of sand and 0.12 g (dry weight) of Ponderosa pine leaf litter, which had been ground in a Wiley Mill (Thomas Scientific, Swedesboro, NJ, United States). The microcosms were sterilized by autoclaving (at 121°C and 15 psi) three times for 1 h each, with at least an 8-h resting interval between each autoclave cycle. Microbial communities were extracted from soil samples (*n* = 206) on the day of inoculation by suspending 1 g of soil in 9 ml of phosphate-buffered saline (PBS, pH 7.4), then creating a 1000-fold dilution in PBS amended with NH_4_NO_3_ at a final concentration of 4.8 mg⋅ml^–1^. We used a high nitrogen background comparable to levels used in field studies (Mueller et al., 2015) to represent the atmospheric deposition of nitrogen that has already occurred and will continue to increase in natural ecosystems (Galloway et al., 2004). The 44-day microcosm incubation included a 14-day equilibration phase with a small amount of litter intended to activate the communities, followed by a 30-day test phase with a much larger amount of litter. At the beginning of the equilibration phase, three microcosms per soil sample each received 1.3 mls of inoculum, pipetted directly onto a 0.02 g aliquot of pine litter (*n* = 618 microcosms). These microcosms were then sealed with Teflon-lined crimp caps (preventing desiccation) and incubated at 25°C in the dark for 14 days to equilibrate the communities. Four negative control microcosms, used to confirm the efficacy of sterilization, received the same quantities of PBS and NH_4_NO_3_, but no microbial communities. The headspace in each microcosm was evacuated using a vacuum pump on days 3 and 7, and replaced with sterile-filtered air. On day 14, an additional 0.1 g aliquot of litter sterilized by three rounds of autoclaving was added to each microcosm (resulting in a total of 0.12 g litter), and microcosms were re-sealed. The microcosms were incubated at 25°C in the dark for a further 30 days. On days 2, 5, 9, 16, 23, and 30, CO_2_ was measured by gas chromatography using an Agilent Technologies 490 Micro GC (Santa Clara, CA, United States). After each measurement, the headspace air was evacuated with a vacuum pump and replaced with sterile-filtered air.

### Dissolved Organic Carbon (DOC) and Litter Community Sampling

After the 44-day (total) incubation, microcosms were destructively sampled to measure DOC and community composition. DOC extractions were performed using a rapid, gentle procedure to avoid measurement artifacts arising from microbial growth or microbial cell disruption. For each microcosm, 5 ml of sterile deionized water was added, swirled manually for 30 s, then transferred to two 2-ml tubes. The tubes were centrifuged 4 min at 16,400 × *g*. The supernatants were combined and sterilized by filtration through a 0.2 μm filter. The concentration of DOC in each sample was measured on an OI Analytical model 1010 wet oxidation TOC analyzer (Xylem Inc., Rye Brook, NJ, United States), calibrated daily. Following DOC sampling, material (sand and litter) from each microcosm was frozen at −80°C for DNA extraction.

### Bacterial and Fungal Community Taxonomic Profiling

Samples for community profiling were down-selected based on the mean DOC concentration of each set of three replicate microcosms at day 44 (Supplementary Figure S2). The profiled samples represented the two tails of the distribution of DOC concentrations. Ribosomal RNA gene profiles were obtained for original soil samples (*n* = 128) and their corresponding replicate microcosms (*n* = 384). DNA extractions were performed with a DNeasy PowerSoil 96-well plate DNA extraction kit (Qiagen, Hilden, Germany). The standard protocol was used with the following two exceptions: (1) 0.3 g of material (either soil or microcosm samples comprised of mixed sand and plant litter) was used per extraction; (2) bead beating was conducted using a Spex Certiprep 2000 Geno/Grinder (Spex SamplePrep, Metuchen, NJ, United States) for 3 min at 1900 strokes per minute. DNA samples were quantified with an Invitrogen Quant-iT^TM^ dsDNA Assay Kit (Thermo Fisher Scientific, Eugene, OR, United States) on a BioTek Synergy HI Hybrid Reader (Winooski, VT, United States). PCR templates were prepared by diluting an aliquot of each DNA stock in sterile water to 1 ng⋅μl^–1^. The bacterial (and archaeal) 16S rRNA gene (V3-V4 region) was amplified using primers 515f-R806 (Bates et al., 2010). The fungal 28S rRNA gene (D2 hypervariable region) was amplified using the LR22R primer (Mueller et al., 2016) and the reverse LR3 primer (Talbot et al., 2014); this target sequence is amenable to phylogenetic tree construction and provides genus-level resolution equivalent to that provided by internal transcribed spacer sequences (Porras-Alfaro et al., 2014).

A two-step amplification procedure was used based on Mueller et al. (2015), with Phusion Hot Start II High Fidelity DNA polymerase (Thermo Fisher Scientific, Vilnius, Lithuania). In the first PCR, barcoded amplicons were produced over 22 cycles using gene primers flanked by 6 nt barcodes that jointly provided a unique 12-mer barcode for each sample (Gloor et al., 2010). Cycling conditions were 30 s at 98°C, 22 cycles of (98°C for 15 s, 60°C for 30 s, 72°C for 30 s), and a final extension step of 72°C for 5min. The second PCR extended Illumina adapter sequences on the amplicons over 10 cycles. Cycling conditions were 30 s at 98°C, 10 cycles of (98°C for 15 s, 65°C for 30 s, 72°C for 30 s), and a final extension step of 72°C for 5min. Amplicons were cleaned using a MoBio UltraClean PCR clean-up kit (Carlsbad, CA, United States), quantified using the same procedure as for the extracted DNA, and then pooled at a concentration of 10 ng each. The pooled samples were further cleaned and concentrated using the Mobio UltraClean PCR clean-up kit. All clean ups were undertaken as per the manufacturer’s instructions with the following modifications: binding buffer was reduced from 5X to 3X sample volume and DNA was eluted in 50 μl Elution Buffer. DNA quality of the amplicon pool was assessed with a bioanalyzer, concentration was verified by qPCR, and sequencing was performed on an Illumina MiSeq with paired-end 250 bp chemistry at Los Alamos National Laboratory.

Bacterial and fungal sequences were merged with PEAR v 9.6 (Zhang et al., 2014), quality filtered to remove sequences with 1% or more low-quality (q20) bases, and demultiplexed using QIIME (Caporaso et al., 2010) allowing no mismatches to the barcode or primer sequence. Further processing was undertaken with UPARSE (Edgar, 2013). Sequences with an error rate greater than 0.5 were removed, remaining sequences were dereplicated, OTU clustering was performed at 97%, and putative chimeras were identified *de novo* using UCHIME (Edgar et al., 2011). Bacterial and fungal OTUs were classified via the Ribosomal Database Project (RDP) classifier (Wang et al., 2007). OTUs that were not classified as bacteria or fungi with 100% confidence were removed from the dataset. Bacterial OTUs with less than 80% classification confidence at the phylum level were also removed. The omitted data accounted for less than 5% of the total. Of the 128 source soil samples that yielded high or low DOC concentrations in microcosms, 123 of the samples passed sequence quality control and 1,481,601 and 1,741,698 total sequences were obtained for bacteria and fungi respectively. The sequences represented 5595 bacterial OTUs (an average of 409 detected per soil) and 2270 fungal OTUs (an average of 112 detected per soil). From the day-44 microcosm samples representing the high and low DOC cohorts, a total of 9,576,525 sequences from 349 of 384 microcosms that passed quality control were obtained for bacteria and 13,124,107 sequences from 377 microcosms were obtained for fungi. These represented 2,527 bacterial OTUs (an average of 275 detected per microcosm) and 753 fungal OTUs (an average of 47 detected per microcosm).

Sequence data were deposited in the NCBI Sequence Read Archive (PRJNA515766 for the source soils and PRJNA478595 for the day-44 microcosm samples). All other data including OTU tables are available upon request.

### Total Biomass, Fungal, and Bacterial Abundance

The DNA quantity extracted from each sample was used as a proxy for biomass. Fungal and bacterial abundance were separately estimated by quantitative PCR (qPCR) using 18S rRNA gene primers nu-SSU-1196F and nu-SSU-1536R for fungi (Borneman and Hartin, 2000) and 16S rRNA gene primers EUB 338 (Lane, 1991) and EUB 518 (Muyzer et al., 1993) for bacteria as described by Castro et al. (2010). Assays were performed with the Biorad iQ SyBr Green Supermix on a BioRad CFX Connect Real-Time System (BioRad, Hercules, CA, United States). DNA templates were normalized to 1.0 ng⋅μl^–1^. Six-point calibration standards were created by serial dilution of linearized plasmid DNA containing a cloned *Phoma* 18S rRNA gene fragment (for fungi) or genomic DNA from *Burkholderia thailandensis* E264, ATCC 70038 (for bacteria). Melt curves were generated for every run to detect potential false positives.

### DOC Binding Assay

To assess variation in DOC composition (*a.k.a*., quality), the fraction of DOC able to bind to mineral surfaces was measured for one DOC sample replicate from each of the high DOC (*n* = 64) and each of the low DOC (*n* = 64) day-44 communities. Aluminum oxide was used as a representative mineral for DOC binding (Kleber et al., 2015). For each sample, 0.5 ml of DOC was added to 1 ml of sterile water (3X dilution factor) and 0.3 g of aluminum oxide (Al_2_O_3_). Samples were mixed by inversion with a Thermolyne rocker (Barnstead/Thermolyne, Dubuque, IA, United States) for 30 min and then centrifuged at 16,100 × *g* for 5 min. Supernatant was transferred to a new tube and stored at −20°C until DOC quantification on a TOC analyzer. The percentage of bound DOC was calculated as 100% × [1-(DOC_post–binding_ × dilution factor)/DOC_pre–binding_].

### Statistical Analyses

Community composition analyses were performed with rarefied data unless otherwise stated using functions in the vegan package v 2.4-3 (Oksanen et al., 2013). For original soil samples, bacterial communities were rarefied to 1095 sequences per sample, and fungal communities were rarefied to 1385 sequences per sample. For day-44 microcosms, bacterial communities were rarefied to 1023 sequences and fungal communities were rarefied to 2032 sequences. Bacterial and fungal richness [E(S) from rarefaction] and diversity (Shannon–Wiener index) were compared between the high and low DOC cohorts for both original soil and day-44 microcosm samples using one-way ANOVAs. Bray–Curtis dissimilarity matrices for bacterial and fungal communities were computed using log-transformed data to reduce the weight of highly abundant taxa in dissimilarity scores. Non-metric multi-dimensional scaling was used to create ordination plots illustrating the community dissimilarity relationships. A permutational multivariate analysis of variance (PERMANOVA; Andersen, 2001) was performed to assess whether the community composition of high and low DOC cohorts differed. The individual microcosms (day 44) were treated as independent samples in all statistical analyses because the replicates diverged substantially in community composition by the conclusion of the experiment and were therefore considered biologically distinct. Compositional analyses were also run on each set of replicates (set A, set B, set C) independently to confirm that conclusions were consistent irrespective of how replicates were treated (Supplementary Table S3).

To further compare community composition between high and low DOC cohorts, OTU sequences were grouped phylogenetically at the Family level for bacteria and Order level for fungi to assess differential abundance of individual taxa. This analysis was performed for the original soils and day-44 microcosm samples. Family-level comparisons were not made for fungi due to low classification confidence levels. For fungal orders and bacterial families, OTUs were only used that could be phylogenetically assigned with at least a 70% confidence level from the RDP Classifier. Because taxa with low abundance values were sporadically detected among microcosms, further statistical analysis of individual taxa was restricted to the most abundant bacterial families and fungal orders that comprised on average at least 1% of the sequences of either the high or low DOC cohorts. Differences in taxon abundance in high versus low DOC cohorts were compared by *t*-tests.

Correlations between various community features versus DOC concentrations were measured with Pearson’s (univariate) or Mantel (multivariate) tests. For Mantel tests with the original soils, the average day-44 DOC concentrations among each set of three replicate microcosms were used to generate a Euclidean distance matrix for comparison with bacterial and fungal community Bray-Curtis matrices (*ecodist* package; Goslee and Urban, 2007). For day-44 microcosm community samples, DOC concentrations from all microcosms were used to create the distance matrix. Univariate features included fungal abundance (qPCR), bacterial abundance (qPCR), fungal:bacterial ratios, total biomass (measured as total extracted DNA), OTU richness, and Shannon diversity. All statistical analyses were performed using R v3.3.3 (R Core Team, 2017).

### Prediction Models for DOC Concentration

To predict DOC concentration at day-44, logistic regression models were developed using emergent community features measured at day-0 or day-44. Because logistic models use a sigmoid function to generate binary predictions, they are highly appropriate for our experimental design focused on DOC cohorts (low/high). However, we also showed comparable results are obtained when using simple linear regression models with DOC as a continuous variable (Supplementary Tables S5, S6).

The logistic model with day-0 (original soil) community features used seven community features as variables: total biomass, fungal richness, bacterial richness, fungal diversity, bacterial diversity, fungal abundance, and bacterial abundance. To show the predictive power of the logistic regression model, the total data set was partitioned into 1000 unique permutations of training and testing data with 30% of samples reserved for testing. Training data and testing data were partitioned such that the balance of high DOC and low DOC labels in each set was equivalent. For day-44 community data, replicates were included as an informative source of feature variation, but each set of replicates was kept together in either training or testing sets to maintain independence of the two sets. Variables in the training data were standardized to be zero mean with unit variance, and variables in the testing data were similarly scaled using training data statistics. Scikit-learn’s (Fabian Pedregosa et al., 2011) Logistic Regression model was used to fit to the training data using an ‘L2’ penalty, which penalizes the squared magnitude of each regression coefficient. After fitting the model to training data, feature selection was applied using Scikit-learn’s SelectFromModel function to reject features with regression coefficients less than a threshold value of 1e-5. For each set of training data, the Wald statistic (defined as the regression coefficient divided by the standard error of the regression coefficient) was computed to provide a measure of the significance of model variables (Wasserman, 2011). This process was performed for every permutation of training and testing data, with the Wald statistics and prediction accuracy stored after each permutation. The average regression coefficient, Wald statistic and *p*-value for each feature over 1000 permutations of training and testing are reported in Supplementary Table S4.

Based on the Wald statistics (Supplementary Table S4), three features (biomass, fungal richness, and bacterial richness) were down-selected as the most important variables to predict DOC concentration (see Supplementary Tables S5, S6 and Supplementary Figures S5, S6). A second logistic regression model with the reduced set of features measured at day-44 was applied to 1000 permutations of training and testing data as described above (Supplementary Table S4). Statistical significance of model performance on testing data was computed using a *z*-test for the equality of two proportions to compare the proportion of correctly labeled samples using the logistic regression model to the proportion of correctly labeled samples using the null model. Code for data pre-processing, logistic regression and statistical analysis is available online at https://github.com/MunskyGroup/Albright_et_al_2019.

## Results

### Microbial-Driven Variation in Respiration, DOC Quantity, and DOC Quality Was Large

**FIGURE 1:**
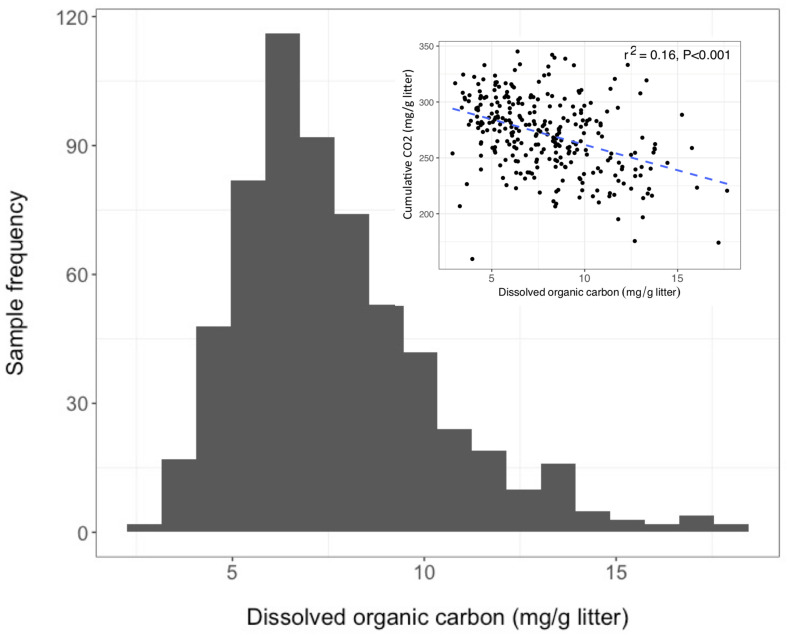
Dissolved organic carbon (DOC) concentrations among 611 microcosms after 44 days of pine litter decomposition. Inset panel - inverse correlation between CO_2_ and DOC.

**FIGURE 2:**
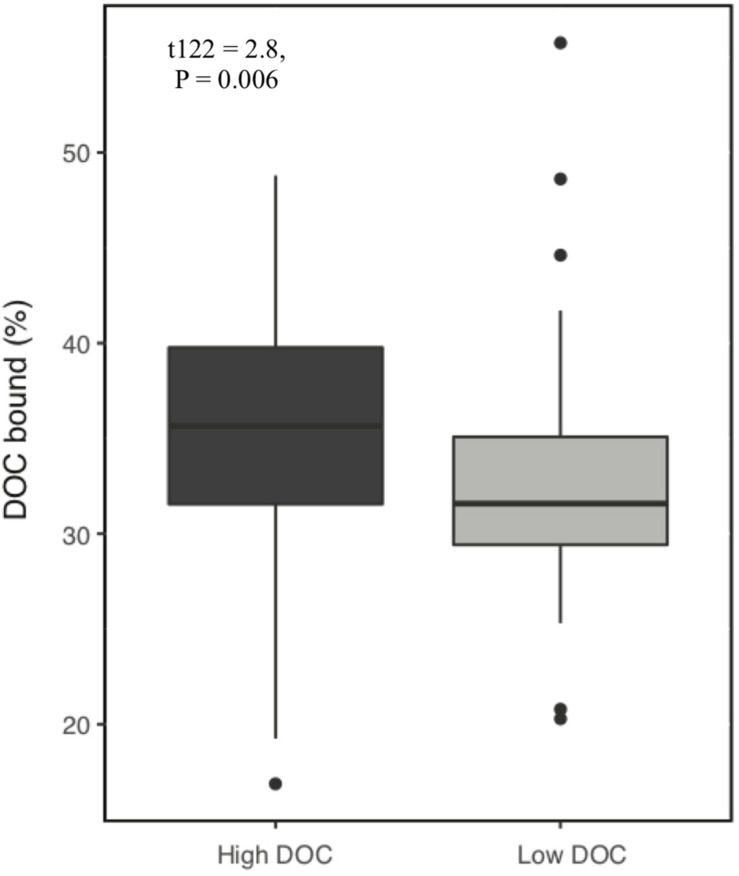
Proportion of dissolved organic carbon (DOC) that binds to aluminum oxide. DOC was obtained from microcosms after 44 days of pine litter decomposition. A greater proportion of DOC binds from high DOC than low DOC samples (*P* = 0.006, *n* = 128).

Over the 6-week decomposition period, cumulative respired CO_2_ varied approximately two-fold between 160 and 345 mg⋅g^–1^ of litter, and DOC concentration varied five-fold between 3 and 18 mg⋅g^–1^ litter (Figure 1). The CO_2_ and DOC from decomposition were negatively and weakly correlated (*r*^2^ = 0.16, *P* < 0.001), showing that cumulative CO_2_ is a poor proxy for DOC. The distribution of mean DOC concentrations among the 206 sets of replicate microcosms was used to delineate two contrasting functional cohorts representing high versus low DOC concentrations (Supplementary Figure S2). The cohorts differed two-fold in mean DOC concentration. DOC concentrations ranged from 3.5 to 6.6 mg g^–1^ litter in the low cohort and from 8.3 to 14.9 mg g^–1^ litter in the high cohort. Although the arbitrarily wide boundaries for each cohort created a risk of impeding discovery of common characteristics within a cohort, the approach was considered a suitable compromise for exploratory analysis, the need for a larger number of samples to support machine learning algorithms, and cost constraints the precluded processing all samples. The cohorts were balanced by requiring each to contain 192 samples (i.e., all three replicate communities derived from 64 source soils). The high and low DOC cohorts varied not only in DOC concentration but also in DOC composition, as indicated by a binding assay with aluminum oxide, a common soil mineral that binds organic carbon (Kleber et al., 2015). The fraction of DOC binding to aluminum oxide ranged from 16.9 to 55.8% among the subset of DOC samples tested. Communities with high concentrations of DOC had, on average, DOC with significantly greater potential for mineral-binding (Figure 2; two-tailed *t-*test, *P* = 0.006).

### Microbial Communities With Contrasting Function Were Geographically Intermingled

The original soils were obtained from eight ecosystem types defined broadly by dominant and minor plant types or by agricultural land-use (Table 1). Ecosystem type significantly influenced the frequency of obtaining a soil community that yielded a high or low DOC concentration in the microcosm experiment (chi-squared test, *P* < 0.001; additional supplemental analyses are available in Albright et al., 2020). However, every ecosystem type except one included soil communities representing both functional cohorts (Table 1), fulfilling the primary objective of acquiring diverse source communities for each DOC cohort. Source soil samples yielding high versus low DOC concentrations in our microcosm study were also geographically intermingled (Supplementary Figure S1) and co-occurred less than 30 m apart at 14% of 49 geographic locations where two or more soil samples were collected from the same site. The seven pairs of co-occurring samples had an average 1.7-fold difference in DOC per pair.

### Community Features Were Linked to DOC Concentration

Community composition, specific taxa, and several emergent community features (biomass abundance, OTU richness, and diversity) were significantly linked to DOC concentration. The links were assessed by comparison of means between the cohorts and/or by correlation with DOC as a continuous variable. Each factor is described in a subsection below.

#### Community Composition

**FIGURE 3:**
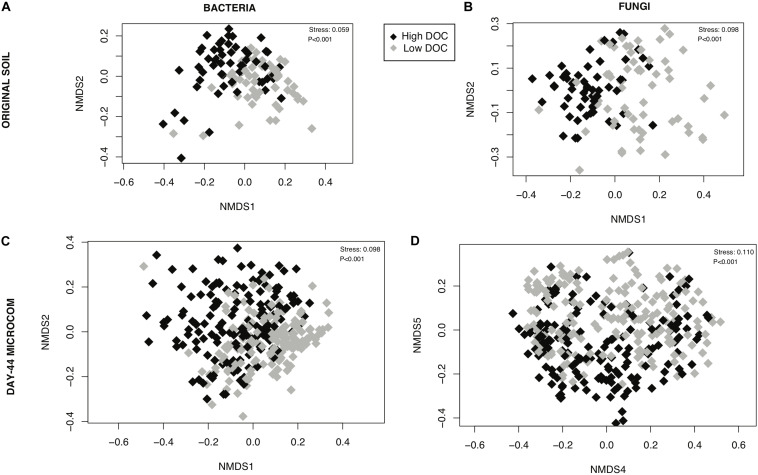
Relationship between microbial community composition and dissolved organic carbon (DOC) concentration. Non-metric multidimensional scaling ordinations performed on rarefied data for **(A)** bacterial communities in original soils, **(B)** fungal communities is original soils, **(C)** bacterial communities in day-44 microcosms **(D)** fungal communities in day-44 microcosms. Points are shaded by DOC cohorts: high (black) and low (gray). The stress value is derived from six dimensions.

The composition of microbial communities in the low versus high DOC cohorts differed significantly, both for the original soil communities and for the day-44 microcosm communities (Figure 3). For the original soil communities and the day-44 microcosm communities, DOC concentration was more strongly correlated with bacterial rather than fungal community composition (Table 2; Mantel test, bacteria *r* = 0.26 or 0.28, *P* = 0.001; fungi *r* = 0.19 or 0.12, *P* = 0.001). Microcosm bacterial communities at day-44 were slightly more correlated with DOC than original communities, while fungal communities showed the opposite trend.

**TABLE 2:** Correlations between DOC concentration and community features.

	Day 0				Day 44			
Feature	DOC correlation (*r*)^*a*^	*P*^*b*^	% Diff of means	*P*	DOC correlation (*r*)	*P*	% Diff of means	*P*
**Bacteria**								
Composition	0.26	**			0.28	**		
Biomass	–0.19	*	36	*	–0.10	NS	19	NS
Diversity	–0.27	**	34	*	–0.55	***	130	***
Richness	–0.37	***	52	**	−**0.64**	***	143	***
**Fungi**								
Composition	0.19	**			0.12	**		
Biomass	–0.14	NS	23	NS	–0.14	NS	36	NS
Diversity	–0.30	***	33	NS	–0.02	NS	3	NS
Richness	−**0.46**	***	69	***	–0.08	NS	14	NS
**Total**								
Biomass^*c*^	–0.28	**	60	***	–0.22	***	47	***
F:B	0.03	NS	−8	NS	0.03	NS	−7	NS

^*a*^The correlation value is Pearson’s rho. The correlation between DOC and community composition was determined with a Mantel test. The highest correlation value at each time point is in bold. ^*b*^*P*-value from significance test. NS, not significant, *<0.5, **<0.01, ***<0.001. ^*c*^Total biomass was measured as total extracted DNA, and therefore, results differ from bacterial and fungal biomass, which were each measured by qPCR assays.

#### Specific Taxa

**FIGURE 4:**
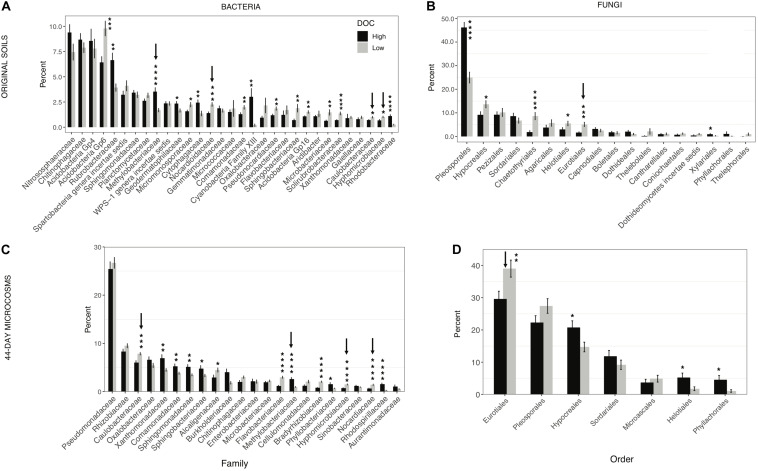
Microbial community composition in high and low dissolved organic carbon (DOC) microcosms. The mean proportion of sequences obtained (±SEM) for each phylogenetic group in the high and low DOC cohorts is shown. **(A)** Bacterial families and **(B)** fungal orders in original soils. **(C,D)** Bacterial families and fungal orders in 44-day microcosms. Only families and orders with a minimum mean relative abundance of 1% in at least one of the DOC cohorts are shown. Statistically significant differences were determined by two tailed *t-*tests (*P < 0.05, **P < 0.01, ***P < 0.001, ****P < 0.0001). Arrows indicate taxa with consistent significant differences between the high and low DOC cohorts with both the original soil data and the 44-day microcosm data.

In the original soil samples and in the day-44 microcosms respectively, 17 of 31 bacterial families and 13 of 23 bacterial families comprising on average at least 1% of the sequences were significantly different in relative abundance between high and low DOC cohorts (Figures 4A,C). Among these families, only four (*Methylobacteriaceae*, *Nocardioidaceae*, *Hyphomicrobiaceae*, and *Caulobacteraceae*) showed consistent differences between DOC cohorts in both the original soils and the day-44 communities (Figures 4A,C). Among the fungal orders comprising on average at least 1% of sequences, 6 of 18 orders in the original soils and 4 of 7 orders in day-44 microcosms were significantly different in relative abundance between high and low DOC cohorts (Figures 4B,D). *Eurotiales* was the only fungal order that was significantly different (higher in the low DOC cohort) between DOC cohorts in both original and day-44 communities.

#### Biomass

The average copy number of bacterial and fungal *rrn* genes determined by qPCR was 108 (bacteria) and 10^7^ (fungi) in the original soil samples as well as in day-44 microcosm samples (Supplementary Table S2). The correlation between total biomass (extracted DNA) and bacterial abundance was similar for soils and microcosms (*r* = 0.69 and 0.66, respectively), whereas the correlation with fungal abundance was weak in microcosm samples (*r* = 0.52 and 0.28, respectively; Supplementary Table S2). Original soil communities in the high DOC cohort had, on average, 36% less biomass than those in the low DOC cohort (Table 2, Supplementary Figure S3; *t*-test *P* < 0.001). Similarly, day-44 microcosm communities in the high DOC cohort had 18% less biomass than those in the low DOC cohort (Table 2, Supplementary Figure S3; *t*-test *P* < 0.001). Even so, DOC was only weakly correlated with biomass (Table 2; *r* = −0.28 or −0.22, *P* ≤ 0.001).

#### Community Richness and Diversity

Microbial community richness [calculated by rarefaction as E(S)] and Shannon diversity were the most significant features linked to DOC concentration (Table 2). Bacterial richness and diversity of the original soil and day-44 microcosm communities were significantly lower in the high compared to the low DOC cohorts (Supplementary Figure S4; *t*-test, richness *P* < 0.002 and diversity *P* ≤ 0.02; also Supplementary Table S3). In both original soils and day-44 microcosms bacterial richness was negatively correlated with DOC concentration and was the community level trait most strongly linked to DOC in day-44 communities (Table 2; Pearson correlation, original soils *r* = −0.39, *P* < 0.001; day-44 microcosms *r* = −0.64, *P* < 0.001). In the original soils fungal richness was also significantly lower in the high DOC cohort (Supplementary Figure S4; two-tailed *t*-test *P* = 0.0001) and negatively correlated with DOC concentration (Table 2; Pearson correlation; *r* = −0.45, *P* < 0.001). No differences in fungal richness were observed in the day-44 microcosms (Supplementary Figure S4; two-tailed *t*-test *P* = 0.187). Fungal diversity did not differ between high and low DOC cohorts in either original soil or day-44 microcosm samples (Supplementary Figure S4; two-tailed *t*-test *P* > 0.05).

### A Classifier Using Community Features Predicted High or Low DOC With Significant Accuracy

**FIGURE 5:**
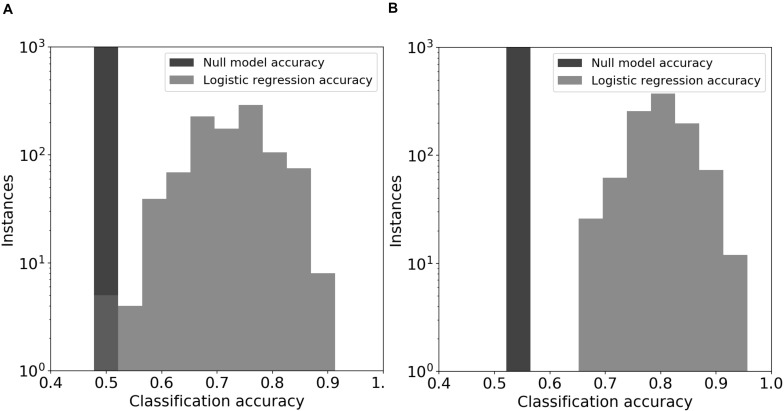
Logistic regression models for DOC. The prediction models used emergent community features from the original soil communities **(panel A)** or the day-44 microcosm communities **(panel B)**. The null model consisted of automatic assignment of samples to the most prevalent DOC cohort that occurred in the test data set. The distributions for the logistic regression models show the prediction accuracy for held-out test data in 1000 permuations of training and test data.

Logistic regression models predicted DOC concentrations (“high” or “low”) in the 44-day microcosms significantly better than chance (Figure 5; *z*-test for a proportion, *P* < 0.05 using day-0 community features and *P* < 0.001 using day-44 community features). The average DOC prediction accuracy of the logistic model from 1000 permutations of training and test data was 0.72 and 0.80, when using feature values from the original soil communities and the final microcosm communities, respectively. In every permutation of training and testing data, the logistic regression model achieved greater prediction accuracy than the null model. In models using original soil community data, the feature importance (Wald statistic) of total biomass, fungal richness, and bacterial richness was −2.5, −1.5, and −1.0, respectively (Supplementary Table S4). In contrast, the importance scores in models using day-44 microcosm community data were −4.5, −0.7, and −6.6 (Supplementary Table S4).

## Discussion

Discovering microbial community features that drive large variation in soil carbon abundance independent of environmental conditions may improve soil carbon modeling and management. Up to 70-fold variation in CO_2_ flux or litter mass loss has been observed in year-long field studies of litter decomposition, and abiotic variables failed to explain the majority of variance (Bradford et al., 2014, 2017). Given the magnitude of unexplained variation in field decomposition studies and in model predictions of soil organic carbon abundance (Wang et al., 2017; Wieder et al., 2018), deciphering the role of microbial community composition is a priority. In our study, we made two important findings: (1) we identified specific community-level features linked to DOC concentration, and (2) we showed the features have strong predictive power when measured before community succession and decomposition begin.

Holding the environment constant within laboratory microcosms while varying microbial community composition reveals an indisputable link between microbial community composition and decomposition outcomes. We built upon valuable prior work by reducing geochemistry as a confounding factor (Strickland et al., 2009) and by using natural microbial source communities instead of isolate mixtures (Matulich and Martiny, 2015). Moreover, we focused on community features driving DOC variation—a priority which has previously been neglected (de Graaff et al., 2015). In our study, high versus low DOC cohorts differed significantly in microbial community composition (Figures 3, 4). The significant difference occurred among the native soil communities as well as among the decomposer communities that arose in the microcosms, demonstrating ecological succession and carbon flow in the laboratory microcosms were constrained by the historical state of the communities in soil. DOC concentration correlated more strongly with the *initial* (original soil) fungal community composition than the *final* fungal community composition (day-44 microcosms) while bacterial community composition showed the opposite trend. Fungi are generally considered the main microbial drivers of plant litter decomposition due to their production of powerful enzymes for deconstruction of plant lignocellulose (Baldrian, 2017). However, bacterial communities also contribute to decomposition outcomes (Glassman et al., 2018). Our results are consistent with the view that fungi are critical in launching major deconstruction of litter and driving the overall rate, while bacteria play an increasing role over time as secondary consumers shaping the quantity and quality of DOC that remains available for transport into soil.

The large range of variation in CO_2_ and DOC in our study combined with the general magnitude (*c.a.* 75 Pg globally) of natural CO_2_ flux from soil microbial respiration (Schlesinger and Andrews, 2000; Ryan and Law, 2005) supports the concept of steering soil microbial respiration to offset anthropogenic CO_2_ emissions for climate change mitigation (Gao et al., 2017). The true range in CO_2_ or DOC flux that can arise from manipulating microbial community variation within a natural ecosystem remains unknown. Variation in surface litter carbon flow may be counter-balanced in nature by compensatory processes over longer time-scales (Glassman et al., 2018) or in other components of the carbon cycle, such that an ecosystem will exhibit a fairly stable mean CO_2_ flux. Nonetheless, our findings motivate further investigation of the potential to alter carbon flow over long time scales by manipulating microbial community composition.

The five-fold range we observed in DOC concentration suggests a potential for microbial community control over soil carbon abundance. In natural systems, DOC from surface litter contributes substantially to soil carbon stocks (Kalbitz and Kaiser, 2008). When DOC from decomposing surface litter is transported to deeper layers, some of the carbon adsorbs to mineral surfaces (Kaiser and Kalbitz, 2012; Newcomb et al., 2017) enabling carbon stabilization over millennial timescales (Schoning and Kogel-Knabner, 2006; Rumpel and Kogel-Knabner, 2011). Because the amount of carbon stored is related to the magnitude of DOC flux (Kalbitz and Kaiser, 2008), microbial communities that yield larger quantities of DOC create a possibility for greater soil carbon storage.

Our results show that microbial community composition also alters DOC quality, which plays a role in soil carbon accumulation. Communities with high concentrations of DOC had, on average, DOC with higher mineral binding potential. Enrichment of DOC with compounds that have greater affinity for mineral surfaces can increase carbon stabilization in soil (Kleber et al., 2015). Enrichment of DOC may occur through different mechanisms including (a) variable depletion of compounds released from plant litter, (b) production of taxon-specific microbial by-products (e.g., polyphenolics produced by Actinobacteria) (Trigo and Ball, 1994) and (c) release of taxon-specific residues from dead microbial cells such as melanin, chitin, B-glucans, or glycoproteins (e.g., glomalin) from fungi (Kogel-Knabner, 2002; Fenandez and Koide, 2012; Siletti et al., 2017). Combining the effects of DOC quantity and quality (i.e., mineral binding capacity), we observed a seven-fold range in the quantity of carbon that could be readily stabilized in soils. In a natural ecosystem, the *realized* quantity of carbon stored would depend on additional factors such as the magnitude of precipitation events for DOC transport to deep mineral layers (Neff and Asner, 2001), soil porosity (Bailey et al., 2017), soil mineralogy and chemistry (Doetterl et al., 2015), and variation in the composition of subsurface microbial communities that control the extent of DOC decomposition during DOC transport through the soil (Dong et al., 2017).

Identifying specific community features that drive decomposition outcomes is a crucial advance beyond demonstrating a basic link between community composition and outcomes. *Eurotiales*, *Nocardioidaceae*, *Hyphomicrobiaceae*, *Caulobacteraceae*, and *Methylobacteriaceae* were strongly linked to the DOC cohorts based on their consistent, significant differences in relative abundance between cohorts in samples from the original soils and day 44 microcosms (Figure 4). Members of these taxonomic groups are early to mid-stage decomposers of plant litter as well as fungal necromass (Baldrian et al., 2012; Matulich et al., 2015; Brabcová et al., 2016; Kielak et al., 2016; Purahong et al., 2016; Bonanomi et al., 2018; Bani et al., 2019; Sauvadet et al., 2019; Wilhelm et al., 2019; Kong et al., 2020). The groups represent a mix of generalists (*Eurotiales*, *Nocardioidaceae*) and specialists with noteworthy physiological characteristics (*Hyphomicrobiaceae*, *Caulobacteraceae*, and *Methylobacteriaceae*). *Methylobacteriaceae* is a family of obligate aerobes that consume C1 to C4 compounds (Eberspächer, 2015). This family was more abundant in the high DOC cohort. The most prominent genus in our study was *Microvirga* (Supplementary Table S1). Given the narrow substrate range of *Methylobacteriaceae*, their importance in shaping DOC concentrations is puzzling. *Hyphomicrobiaceae* and *Caulobacteraceae* are known for oligotrophy. The most relevant genera in our study were *Devosia*, *Hyphomicrobium*, *Caulobacter*, and *Phenylobacterium* (Supplementary Table S1). The elevated abundance of *Hyphomicrobiaceae* and *Caulobacteraceae* in the low DOC cohort suggests they may reduce the concentration of some DOC compounds to growth-limiting levels. When substrates are growth-limiting, the average carbon use efficiency of a community may decline as more taxa invest in production of extracellular enzymes to acquire resources (Malik et al., 2019; Ramin and Allison, 2019), and the range of variation among taxa in carbon use efficiency decreases (Saifuddin et al., 2019). If such a shift occurs, communities in the low DOC cohort may have lower carbon storage potential owing to a decreased efficiency of biomass production (Six et al., 2006), in addition to having a lower quantity and quality of DOC for mineral stabilization. Based on these observations, the use of an oligotroph–copiotroph trait axis for soil carbon modeling (Wieder et al., 2015b) merits further consideration. However, other distinctive physiological characteristics such as predation and antibiotic antagonism have also been linked to the DOC cohorts and may be of equal or greater importance (Albright et al., 2020).

Since regional and global carbon models cannot account for thousands of different microbial species’ abundances, we focused on emergent community properties as features that may predict DOC. Among the seven features we examined, total biomass, fungal richness, and bacterial richness were the most important features linked to DOC concentration (Table 2 and Supplementary Tables S3, S4). Although DNA is a crude biomass proxy affected by variation within and among fungal species in the ratio of genome-mass-to-cell-size and in extraction efficiency, it is the most economical proxy for widespread use when other community composition features will also be measured by DNA sequencing. The predictive power of biomass, fungal richness, and bacterial richness was robust, as indicated by the nearly equal performance of the set of features measured before or after 6 weeks of community succession. The reversal in the importance of fungal versus bacterial richness as DOC predictive features at the beginning versus end of the microcosm incubation again points to time-dependent roles of fungi and bacteria in the decomposition process that merit further investigation. The importance of initial fungal taxon richness suggests fungi may create early priority effects that constrain the trajectory of decomposition and shape the assembly of bacterial communities that ultimately control DOC concentration and composition. The capacity to use easily measured community features to forecast the functional patterns of soil communities can simplify mapping the geographic distribution of a functional pattern that is driven by microbes, not the environment. To be climate relevant, an unexpected microbial functional pattern must be geographically prevalent to cause the mean behavior of an ecosystem to deviate from conventional soil carbon models. Our predictive DOC model is an encouraging first step toward a capability to assess geographic prevalence. However, considerable validation of the predictive model is needed, including confirmation of prediction performance when applied to new soils and when applied to other litter types.

The strong correlation between lower bacterial richness and higher DOC concentration is a priority for further analysis. If bacterial richness proves to be a robust factor to predict DOC concentration among natural ecosystems, understanding the factors that control richness may reveal mechanisms that can be used to improve prediction or management of soil carbon dynamics. Bacterial richness is known to vary at the landscape scale, declining with greater aridity (Maestre et al., 2013; Tu et al., 2017) and with lower pH (Bahram et al., 2018). However, richness that is strongly driven by environmental factors may be uninformative in soil carbon models because the empirically calibrated environmental variables in conventional models are likely to capture the linked functional consequences. Biotic interactions that affect species richness independent of the environment are more likely to create unexplained variance in soil carbon models. Biotic interactions that reduce richness and suppress function may include antibiotic production (Frey-Klett et al., 2011), predation (Sockett, 2009), or bacteriophage activity (Williamson et al., 2017). Evidence for these phenomena in our microcosm study are described in Albright et al. (2020).

## Conclusion

To improve climate predictions by including microbial processes in soil carbon models, climate-relevant microbial processes and simple features that represent them must first be identified, as has been achieved with plant traits (Laughlin, 2014; Wieder et al., 2015b). Our study showed a strong influence of microbial community composition over decomposition outcomes in a constant environment, resulting in large differences in carbon flow from litter decomposition. It is reasonable to expect that microbial composition drives variation in every component of soil carbon cycling (e.g., surface litter decomposition, subsurface litter decomposition, plant productivity and carbon allocation). Our findings motivate investigation of this phenomenon in natural systems to assess its importance to climate feedbacks within and among existing ecosystems and its implications for managing soil carbon. We identified a high-level feature—bacterial richness—linked to DOC concentration and known to be geographically patterned. Bacterial richness has also been linked to carbon fate in mammals where lower richness correlates with increased carbon storage in the host (Le Chatelier et al., 2013; Shabat et al., 2016). Our findings raise the tantalizing possibility of discovering robust principles that underpin functional patterns in extremely diverse systems ranging from soils to animal guts.

## Data Availability Statement

The datasets generated for this study can be found in the NCBI Sequence Read Archive (PRJNA515766 for the source soils and PRJNA478595 for the day-44 microcosm samples).

## Author Contributions

JD designed the experiments. RJ, DL, LG-G, and AR conducted the experiments. RJ, LG-G, AR, and TY performed the laboratory measurements. MA, RJ, JT, BM, RM, and AW conducted the data analyses. JD, MA, and RJ drafted the manuscript. MA, MK, RJ, JT, and BM contributed to writing and revisions. All authors read and approved the final version of the manuscript.

## Conflict of Interest

The authors declare that the research was conducted in the absence of any commercial or financial relationships that could be construed as a potential conflict of interest.
