# Supplementary material for: Soil Bacterial and Fungal Richness Forecast Patterns of Early Pine Litter Decomposition
Source: Front Microbiol. 2020 Nov 6;11:542220. doi: 10.3389/fmicb.2020.542220 (PMC7677502; doi:10.3389/fmicb.2020.542220)
Supplement: Supplementary file 1 [file Data_Sheet_1.pdf]

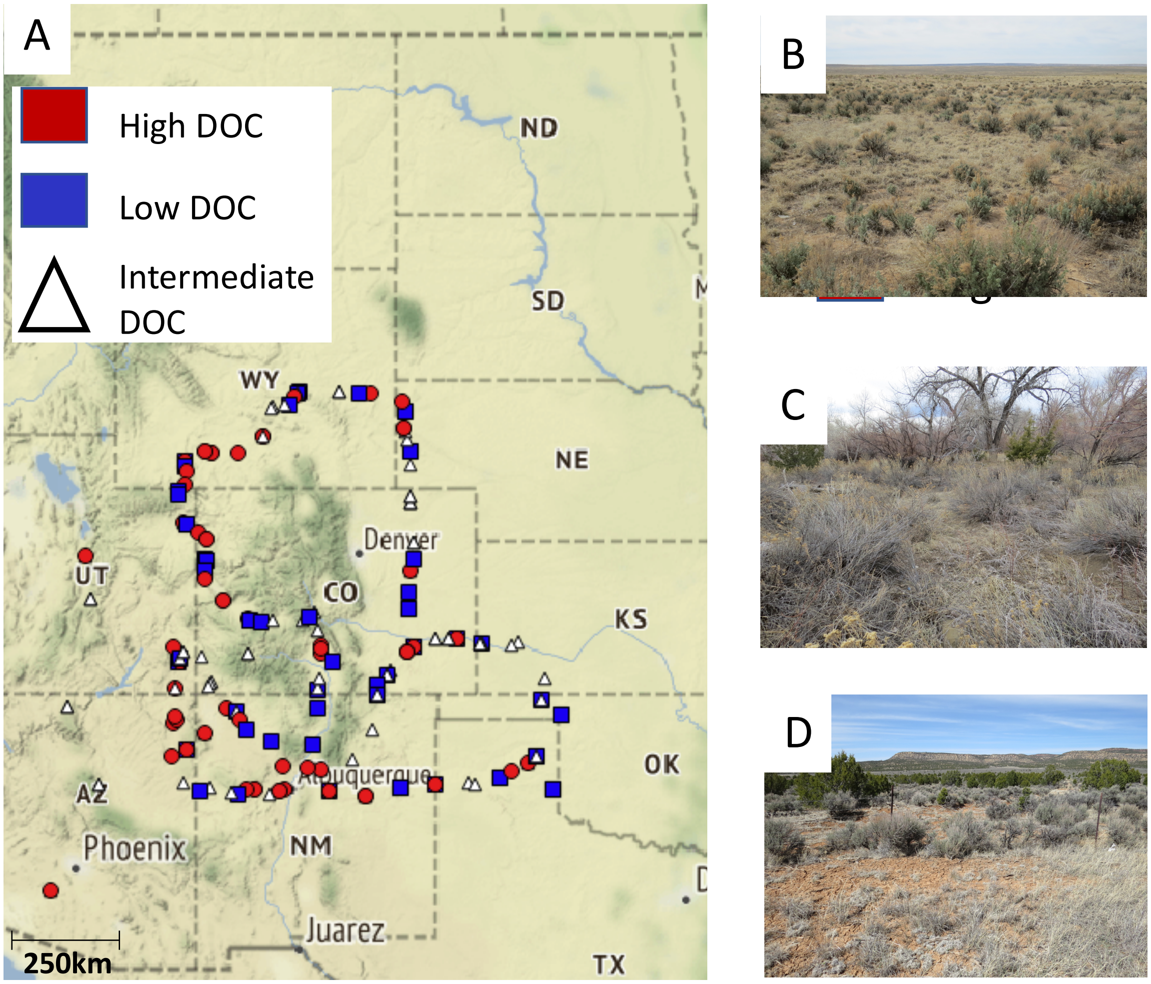

Figure S1. Original soil sampling points in the southwestern United States. (A) Map of sample locations with symbols indicating the cohorts delineated by dissolved organic carbon (DOC) concentration after 44 days of pine litter decomposition in microcosms under constant environmental conditions. Average DOC values for each original soil sample were calculated from the mean of three replicate microcosms at day 44 and grouped in the high, intermediate, or low DOC cohorts. Soils locations were classified as eight ecosystem types, listed in Table 1. Photos show examples of the three most prevalent types: (B) grassland-shrub, (C) mixed, and (D) juniper woodland – grass.

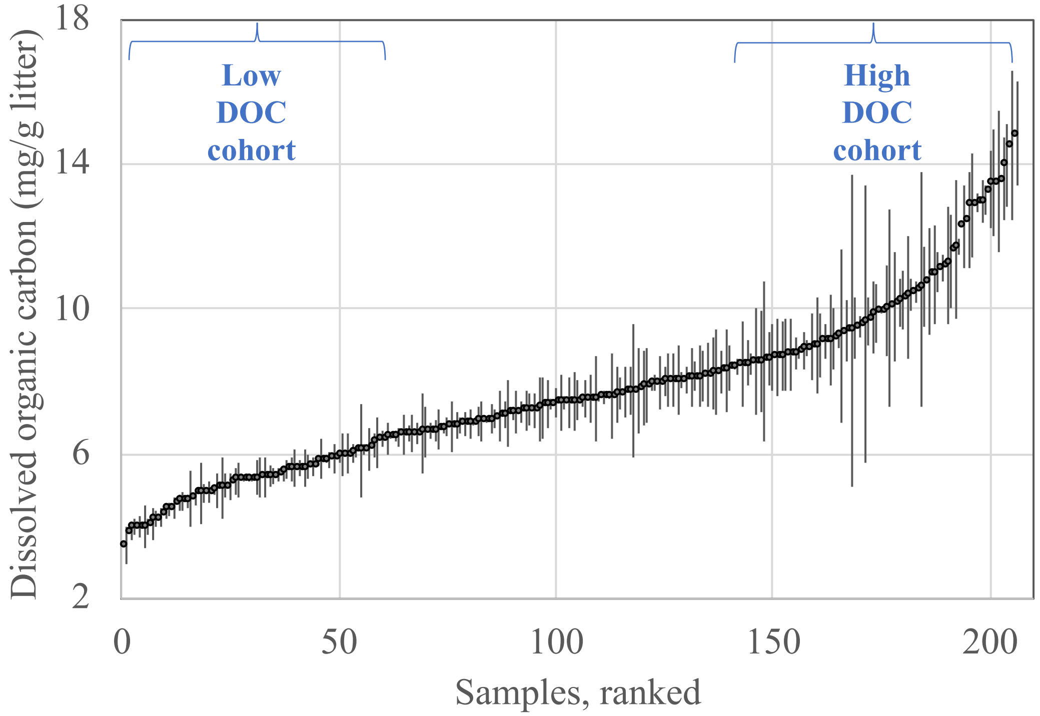

Figure S2. Range of DOC concentrations (ranked low to high) after 44 days of pine litter decomposition by microbial communities from different soils. Each original soil community was inoculated into three replicate microcosms. Points are means of the replicates ± SEM. Community cohorts at ends of the distribution were selected for community profiling.

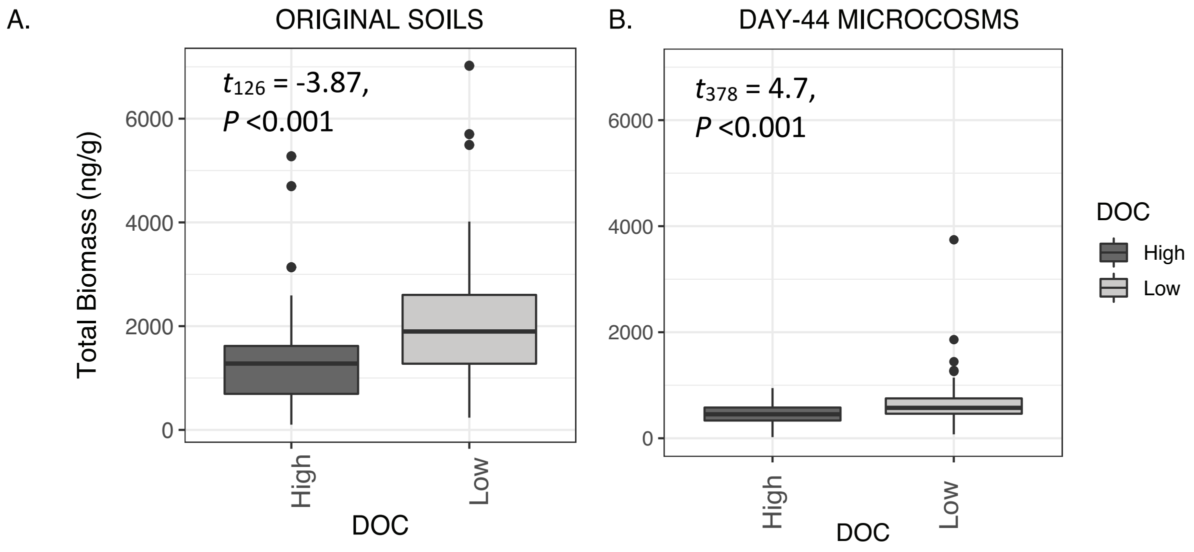

Figure S3. Total biomass in a) original soils and b) day-44 microcosm samples, as measured by DNA quantity in high and low DOC groups. Each box shows the median, the 25% and 75% quartile range, and the result from a 2-tailed t-test.

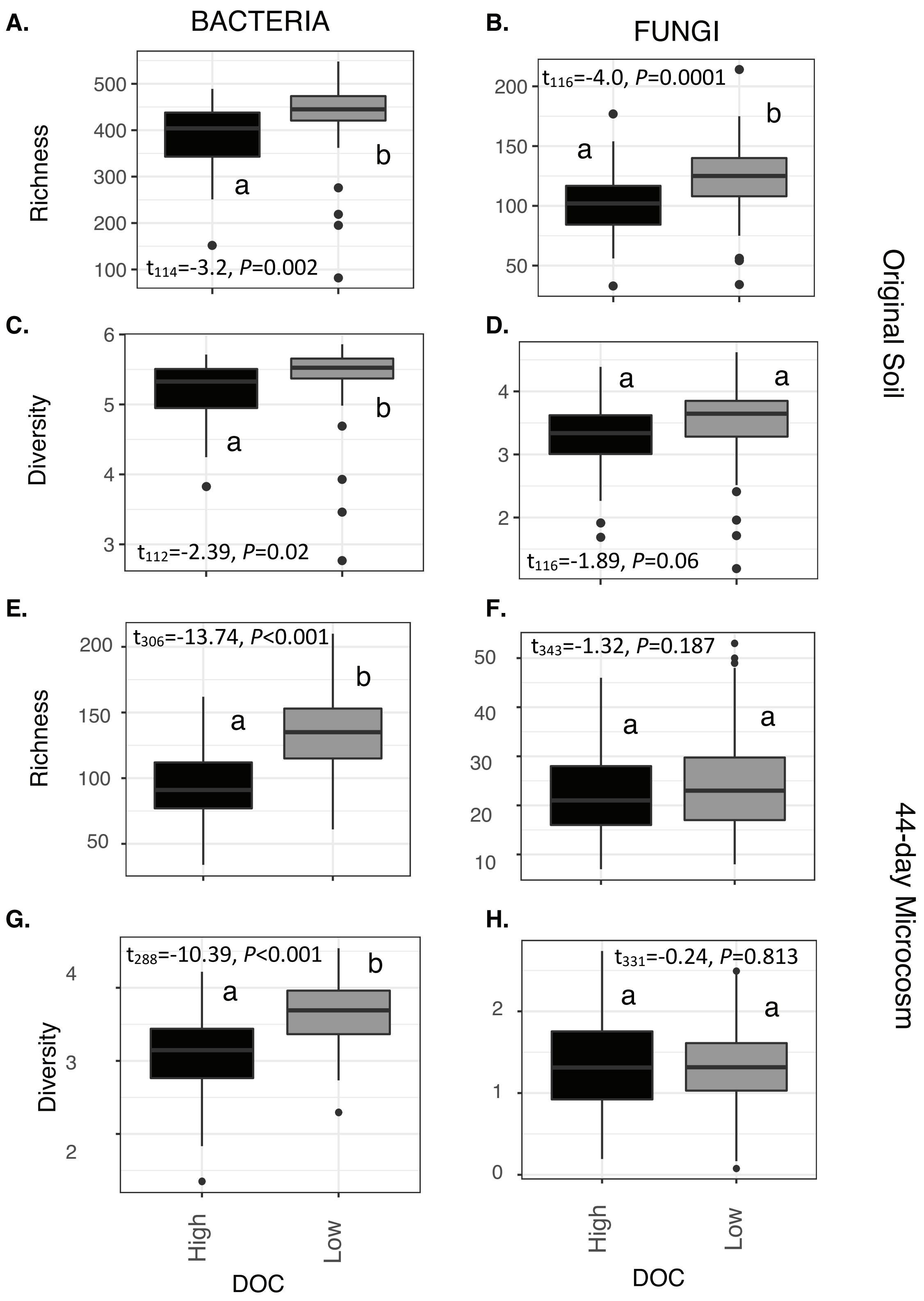

A.
BACTERIA
B.
FUNGI
Richness
t_{114}=-3.2, *P*=0.002
t_{116}=-4.0, *P*=0.0001
C.
D.
Diversity
t_{112}=-2.39, *P*=0.02
t_{116}=-1.89, *P*=0.06
Original Soil
E.
F.
Richness
t_{306}=-13.74, *P*<0.001
t_{343}=-1.32, *P*=0.187
G.
H.
Diversity
t_{288}=-10.39, *P*<0.001
t_{331}=-0.24, *P*=0.813
44-day Microcosm
High
Low
DOC

Figure S4. Microbial diversity statistics for the original soil and the day-44 microcosm communities. Operational taxonomic unit (OTU) richness in original soils for bacteria (A) and fungi (B). Shannon-Wiener diversity for bacteria (C) and fungi (D) in original soils. Operational taxonomic unit (OTU) richness in day-44 microcosms for bacteria (E) and fungi (F). Shannon-Wiener diversity for bacteria (G) and fungi (H) in day-44 microcosms. Each box shows the median value and the 25% and 75% quartile range. Statistics from two-tailed t-tests are shown.

**Genus**	**avg % abundance**		**Sig. OTUs ($P<0.05$)[a]**		**% of Family[b]**		**Genus significance[c]**	
	day 0	day 44	day 0	day 44	day 0	day 44	day 0	day 44
Eurotiales								
Aspergillus	1.1	29.3	0 of 15	3 of 20	32	87	0.48	0.020
Penicillium	1.8	4.3	4 of 13	1 of 11	53	13	<0.001	0.82
Nocardioidaceae								
Nocardioides	0.9	0.1	7 of 27	3 of 16	48	36	<0.001	0.025
Methylobacteriaceae								
Microvirga	2.1	1.4	4 of 8	6 of 8	82	84	<0.001	<0.001
Caulobacteraceae								
Caulobacter	0.3	2.2	2 of 16	2 of 9	31	34	0.036	<0.001
Phenylobacterium	0.2	2.2	8 of 19	7 of 19	28	33	<0.001	0.43
Hyphomicrobiaceae								
Devosia	0.6	1.0	2 of 14	5 of 10	67	91	0.53	<0.001
Hyphomicrobium	0.1	0.1	4 of 8	3 of 8	10	7	<0.001	<0.001

[a]The number of OTUs with significant differences (t-tests) between high and low DOC cohorts is listed with the total number of OTUs detected in each genus.
[b]The percentages refer to the contribution of the Genus to the observed Family abundance calculated as: 100% x (Genus average abundance)/(Family average abundance).
[c]The p-values are from t-tests of genus abundance values between high and low DOC cohorts.

Table S2. Correlation of qPCR fungal and bacterial abundance (adjusted to copies·g^{-1} soil) with total DNA

	Soil (day 0)		Microcosm (day 44)	
	Avg. abundance[a]	Pearson's rho	Avg. abundance[a]	Pearson's rho
Bacteria	2.1E+08	0.69	3.5E+08	0.66
Fungi	2.7E+07	0.52	9.3E+07	0.28

[a]The abundance values are estimated copy numbers of the bacterial 16S rRNA gene and fungal LSU rRNA gene.

Table S3. Comparison of high and low DOC cohorts by replicate sets at day 44.

Factor	Replicate	Test	df	Statistic	p value
Biomass	Set A	t-test, 2-tailed	102	-2.814	0.006
	Set B	t-test, 2-tailed	98	-2.788	0.006
	Set C	t-test, 2-tailed	96	-2.535	0.013
Bact. Composition	Set A	permanova	1		0.001
	Set B	permanova	1		0.001
	Set C	permanova	1		0.001
Fungal Composition	Set A	permanova	1		0.003
	Set B	permanova	1		0.003
	Set C	permanova	1		0.008
Bact. Richness*	Set A	t-test, 2-tailed	102	-7.471	3E-11
	Set B	t-test, 2-tailed	99	-8.712	7E-14
	Set C	t-test, 2-tailed	97	-6.731	1E-09
Bact. Diversity*	Set A	t-test, 2-tailed	102	-5.923	4E-08
	Set B	t-test, 2-tailed	98	-6.571	2E-09
	Set C	t-test, 2-tailed	96	-5.820	8E-08
Fung Richness	Set A	t-test, 2-tailed	109	-0.459	N.S.; 0.65
	Set B	t-test, 2-tailed	116	-1.750	N.S.; 0.08
	Set C	t-test, 2-tailed	114	-0.243	N.S.; 0.81
Fung Diversity	Set A	t-test, 2-tailed	109	-0.777	N.S.; 0.44
	Set B	t-test, 2-tailed	116	-0.607	N.S.; 0.54
	Set C	t-test, 2-tailed	114	1.016	N.S.; 0.31

*Bacterial richness and diversity for each set of replicates were also significantly different between high and low DOC cohorts when tested with 255 community profiles rarefied to 5036 sequences each, instead of 1023. P values ranged from 10^{-10} to 10^{-12} for richness and 10^{-7} to 10^{-8} for diversity.

Logistic Regression Models Predicting day 44 DOC Concentrations

Logistic regression models using day-0 or day 44 community features to predict day-44 DOC were created as described in Methods. The model constructed with Day 0 features used seven emergent community features as variables. The model constructed with Day 44 features used only the top three most significant day-0 community features from the day-0 model (i.e., those features with a Wald statistic p value less than 0.4). The average regression coefficient, Wald statistic and p-value for each feature over 1000 permutations of training and testing are reported in Table S4. The overall average day-0 prediction accuracy for the two models was 0.724 and 0.8, respectively (Figure 5).

Table S4. Logistic regression model - average regression coefficients, Wald statistics, and P values of community features from 1000 random permutations of training and testing data.

	**Day 0 Model**			**Day 44 Model**		
Feature	Avg Coefficient	Avg Wald stat.	P value	Avg Coefficient	Avg Wald stat.	P value
Total Biomass	-1.01637	-2.47405	0.013	-1.269105	-4.48237	7.4E-06
Fung Abundance	-0.007928	-0.01441	0.989			
Bact Abundance	0.0003	0.00153	0.999			
Fung Richness	-0.546487	-1.53172	0.126	-0.147888	-0.69788	0.049
Fung Diversity	0.001941	0.00411	0.997			
Bact Richness	-0.804171	-0.9644	0.335	-2.244865	-6.63885	3.2E-11
Bact Diversity	0.702019	0.75927	0.448			

Note: The sign of the Wald statistic indicates a positive or negative contribution to DOC abundance, and the magnitude of the *absolute* value reflects the degree of importance.

Logistic Regression Analysis Using Entire Data Set

As an added confirmation that our final regression models were significant, we combined all training and testing data and computed the McFadden pseudo R^2 value and log likelihood ratio P-values, similar to the conventional approach used by others (e.g., Maynard et al., 2018). These tests revealed an R^2 of 0.292 for the 7-feature Day 0 model (P = 1.22E-7) and R^2 of 0.465 for the 3-feature Day 44 model (P = 7.63E-39). Both analyses were in agreement for the most significant features: (Total Biomass, Fungal Richness, Bacterial Richness, Bacterial Diversity) for Day 0 features, and (Bacterial Richness and Total Biomass) for Day 44 features. The pseudo R_2 values and LLR P values reflect the goodness of fit of the model to the entire data set. In contrast, the more modest P values reported in the main text represent the significance of model predictions on held-out testing data.

Pseudo-replication, Bacterial Diversity, and Linear Regression

Additional modeling was performed to address the following three topics:

Pseudo-replication - The model results in Figure 5B used data from microcosm replicates because the variation among replicates was informative, but this creates a concern about inflation of statistical significance. To address this concern, the average feature values from

each set of replicates were computed, thereby eliminating replicates and making the number of samples comparable to the number used in the day 0 model. Prediction results were similar for models with or without the use of replicates (Figure S5).

Bacterial diversity as a day-44 model feature – To confirm the validity of excluding bacterial diversity as a feature in the day-44 model reported in Figure 5, we repeated the modeling with inclusion of bacterial diversity (Tables S5, S6) and coupled it with a sensitivity analysis that confirmed the validity of excluding the feature (Figure S6).

Logistic versus linear regression – Although logistic models were the most appropriate for our experimental design focused on two DOC cohorts, we strengthened the findings by confirming that linear regression models treating DOC as a continuous variable provide comparable results as shown below (Table S6).

Table S5. Logistic regression model - average regression coefficients, Wald statistics, and P values of community features from 1000 random permutations of training and testing data.

	Day 44 Model with replicates			Day 44 Model, replicates averaged		
Feature	Avg Coefficient	Avg Wald stat.	P value	Avg Coefficient	Avg Wald stat.	P value
Total Biomass	-1.261446	-4.46178	0.8E-6	-1.218004	-3.03749	0.002
Fung Richness	-0.158716	-0.74523	0.456	-0.134121	-0.38267	0.702
Bact Richness	-2.423591	-4.32994	1.5E-5	-1.988159	-1.95476	0.051
Bact Diversity	0.1958448	0.43728	0.662	-0.250452	-0.25455	0.799

Figure S5. Illustration of prediction performance of logistic models using day-44 features summarized in Table S4. (A) Model using replicates. (B) Model with replicates averaged.

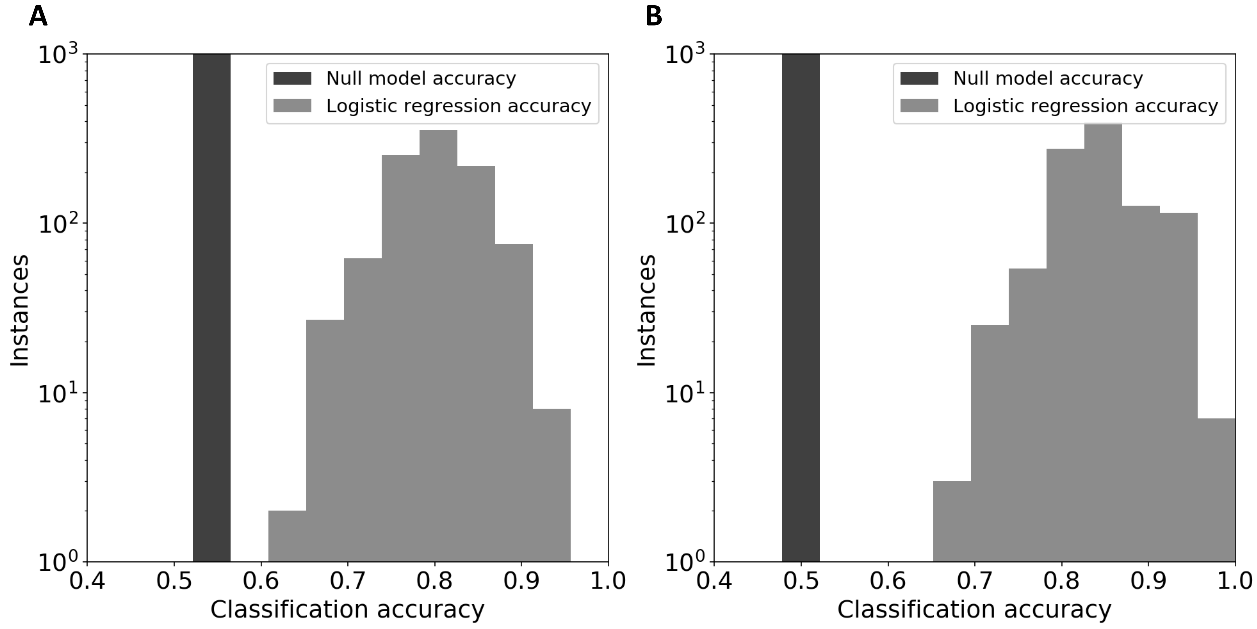

Linear regression models using day-0 or day 44 community features to predict day-44 DOC were created as described for logistic models in Methods, except that features from day-44 microcosm replicates were averaged prior to modeling to avoid concerns about pseudo-replication. The correlation coefficient and p-value of the regressions were computed using Scipy's (Virtanen, 2020) linregress function, which assigns a p-value for a hypothesis test that compares to a null model of only the regression coefficient (slope is zero). The regressions of true DOC versus the averaged out-of-bag predictions of DOC using a linear regression model were significant (day 0 features: r = 0.506, P = 1.52e-8; day-44 features: r = 0.508, P = 4.46e-9).

Both the logistic regression and linear regression models found the same day-0 features to be unimportant (fungal abundance, bacterial abundance, fungal diversity all had p-values > .95; Table S4, S6). A difference between the logit and the linear regression model was the importance placed on total biomass. While the logistic regression model found total biomass to be an important day-44 feature for predicting DOC, the linear regression model improved when total biomass was dropped (Figure S6). Both models showed that including fungal richness and bacterial richness improved prediction performance of DOC (Tables S4,S5, S6; Figure S6). Training and testing the logistic and linear regression models after dropping each day-44 feature showed that model performance is improved after dropping bacterial diversity (Figure S6).

Table S6. Linear regression model - average regression coefficients, Wald statistics, and P values of community features from 1000 random permutations of training and testing.

	**Day 0 Model**			**Day 44 Model**		
Feature	Avg Coefficient	Avg Wald stat.	P value	Avg Coefficient	Avg Wald stat.	P value
Total Biomass	-0.055273	-2.47405	0.568	-0.111849	-1.17661	0.239
Fung Abundance	-0.000238	-0.00146	0.999			
Bact Abundance	0.000979	0.007607	0.994			
Fung Richness	-0.219131	-2.02577	0.043	-0.134087	-1.38802	0.165
Fung Diversity	-0.000134	-0.00085	0.999			
Bact Richness	-1.230771	-3.55047	3.9E-04	-0.643199	-2.66445	0.008
Bact Diversity	1.018973	2.89029	3.8E-03	0.172369	0.722764	0.470

To probe the importance of day-44 features, the logistic and linear regression models were trained and tested after dropping each day-44 community feature (total biomass, fungal richness, bacterial richness, and bacterial diversity). Prediction performance was determined based on out-of-bag predictions after bootstrapping the data set over 1000 permutations with 30% of the data withheld for testing in each permutation.

Figure S6. Feature Dropout analysis of Day-44 community features. (A) Logistic model. (B) Linear regression model.

A

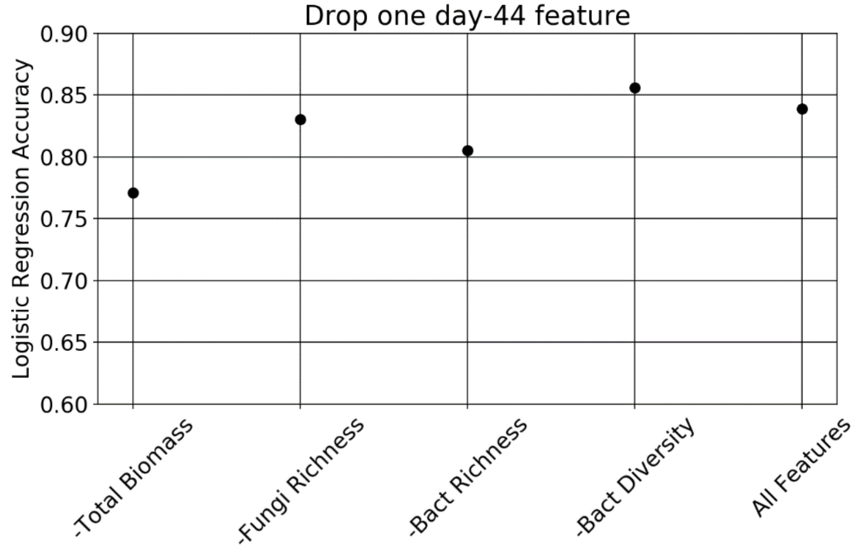

B

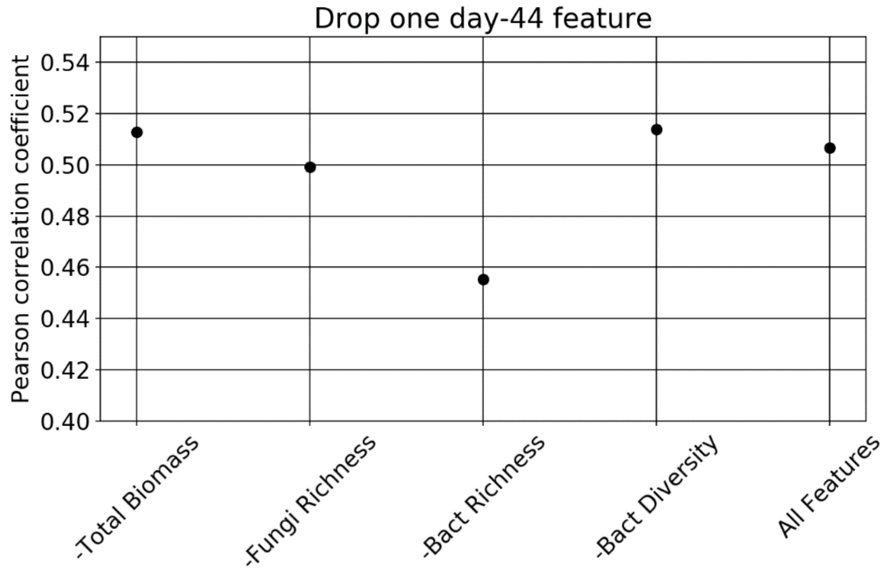

Citations

Virtanen et al. SciPy 1.0: Fundamental Algorithms for Scientific Computing in Python. Nature Methods, 2020.

Maynard et al. *Species associations overwhelm abiotic conditions to dictate the structure and function of wood-decay fungal communities*. Ecology, 99(4), 2018, pp. 801–811
